# Successful Establishment of the First Neonatal Respiratory Extracorporeal Membrane Oxygenation (ECMO) Program in the Middle East, in Collaboration With Pediatric Services

**DOI:** 10.3389/fped.2020.00506

**Published:** 2020-09-11

**Authors:** Mohammed Elkhwad, Kiran S. More, Dhullipala Anand, Samira Al-Maraghi, Myles Crowe, Duane Wong, Julianne Metcalf, Santosh K. Yadav, David Sigalet

**Affiliations:** ^1^Division of Neonatology, Sidra Medicine, Doha, Qatar; ^2^Weill Cornell Medicine, Doha, Qatar; ^3^Department of Respiratory Therapy, Sidra Medicine, Doha, Qatar; ^4^Division of Cardiac Intensive Care Unit, Sidra Medicine, Doha, Qatar; ^5^Nurse Education Subunit, Sidra Medicine, Doha, Qatar; ^6^Functional and Molecular Imaging, Sidra Medicine, Doha, Qatar; ^7^Department of Pediatric Surgery, Sidra Medicine, Doha, Qatar

**Keywords:** extracorporeal membrane oxygenation, newborn infant, respiratory ECMO, ECMO program, persistent pulmonary hypertension, meconium aspiration, extracorporeal life support

## Abstract

**Background:** Extracorporeal membrane oxygenation (ECMO) is a complex life-saving support for acute cardio-respiratory failure, unresponsive to medical treatment. Starting a new ECMO program requires synergizing different aspects of organizational infrastructures and appropriate extensive training of core team members to deliver the care successfully and safely.

**Objectives:** To describe the process of establishing a new neonatal ECMO program and to evaluate the program by benchmarking the ECMO respiratory outcomes and mechanical complications to the well-established Extracorporeal Life Support Organization (ELSO) registry data.

**Materials and Methods:** We reviewed the processes and steps involved in planning and setting up the new ECMO program. To assess the success of the ECMO implementation program, we retrospectively reviewed data of clinical outcomes and technical complications for the first 11 patients who have received ECMO therapy for respiratory indications since program activation (July 2018–May 2020). We analyzed mechanical complications as a tool to measure infrastructures and our effective training for the core team of ECMO specialists. We also looked at all clinical complications and benchmarked these numbers with the last 10 years of ELSO registry data (2009–2019) in the corresponding categories for comparison. Chi-square test was used to compare, and outcomes are presented in percentage; a *p*-value of <0.05 is considered significant.

**Results:** A total of 27 patients underwent ECMO in the hospital, out of which 11 (six neonatal and five pediatric) patients had acute respiratory failure treated with venovenous (VV) ECMO or veno-arterial (VA) ECMO over a 22-month period. We had a total of 3,360 h of ECMO run with a range from 1 day to 7 weeks on ECMO. Clinical outcomes and mechanical complications are comparable to ELSO registry data (no significant difference); there were no pump failure, oxygenator failure, or pump clots.

**Conclusions:** Establishing the ECMO program involved a multisystem approach with particular attention to the training of ECMO team members. The unified protocols, equipment, and multistep ECMO team training increased staff knowledge, technical skills, and teamwork, allowing the successful development of a neonatal respiratory ECMO program with minimal mechanical complications during ECMO runs, showing a comparable patient flow and mechanical complications.

## Introduction

Extracorporeal life support (ECLS) or extracorporeal membrane oxygenation (ECMO) is a modern rescue modality used to support critically sick newborn infants with cardiorespiratory dysfunction when conventional therapies have failed (1). The Extracorporeal Life Support Organization (ELSO) registry that collects data on ECLS use and outcomes in children and adults recently published summary data of 78,000 patients so far. Of the patients, about 40% (>29,000) had neonatal respiratory failure, of which 74% survived to hospital discharge (2). Meconium aspiration syndrome (MAS) is a major indication to use ECLS in this population with the best survival, followed by congenital diaphragmatic hernia (CDH) and persistent pulmonary hypertension of the newborn (2, 3). Physician's faith in the ECMO as a treatment modality is confounded by a high rate of neurologic complications in neonatal ECMO along with increased mortality, making it very challenging to set up a new ECMO service (4).

Sidra Medicine (Sidra) hospital is built to be a beacon of learning, innovation, and exceptional care. This new hospital is built to be one of the finest and most technologically advanced hospitals in the world. Sidra is also part of a dynamic research and education environment in Qatar, and through strong partnerships with leading institutions around the world, Sidra is creating an intellectual ecosystem to help advance scientific discovery through investment in medical research.

There has been an improvement in health and a decline in overall mortality over the decades as Qatar has invested in the healthcare system (5). A retrospective study looked at maternal and neonatal survival in Qatar for over 35 years (1974–2008). During these 35 years, there was a remarkable decline (*P* < 0.001) in Qatar's neonatal mortality rate from 26.27/1,000 in 1974 to 4.4/1,000 in 2008 (6).

Previously, there was no neonatal respiratory ECMO program in the Middle East. All ECMO services for the neonatal population were geared toward those who cannot come off cardio-pulmonary bypass (CPB) following cardiac surgery. There was a huge need for this program in Qatar and the region. The ECMO program was built in Sidra medicine with rigorous planning and execution over 1 year (2017–2018) and was activated in June 2018. Setting up a new sophisticated technology into the new clinical environment was a challenging process, especially when used in high-intensity settings and high-risk situations, and the potential risks are magnified. This process of establishing a new ECMO program involved tremendous effort and faced many challenges as it was developed over a completely new Greenfield site hospital (7). Collaboration and coordination of healthcare professionals coming from different corners of the world with different practices and diverse social, cultural backgrounds were a big challenge. Various team models were possible, but after we studied our workforce, we chose a model of ECMO specialists as a primary model to free perfusionists to attend cardiothoracic surgery.

The purpose of this paper is to review the processes, challenges, and achievements of building the first neonatal respiratory ECMO program in the Middle East in the newly emerging Qatar healthcare system and compare the clinical finding by benchmarking with ELSO registry data.

## Methods

We reviewed the comprehensive plan that was developed along with various steps and processes involved in the activation of Sidra ECMO program, which can be classified into the following phases:

1. Planning and preparation
  - Institutional commitment
  - Formation of ECMO Steering Committee
  - Need assessment and setting up the scope.
2. Formation of ECMO team
  - Key personnel
  - Staffing design
  - ECMO specialist model.
3. Training of team
4. ECMO program activation and evaluation.

**Figure 1:**
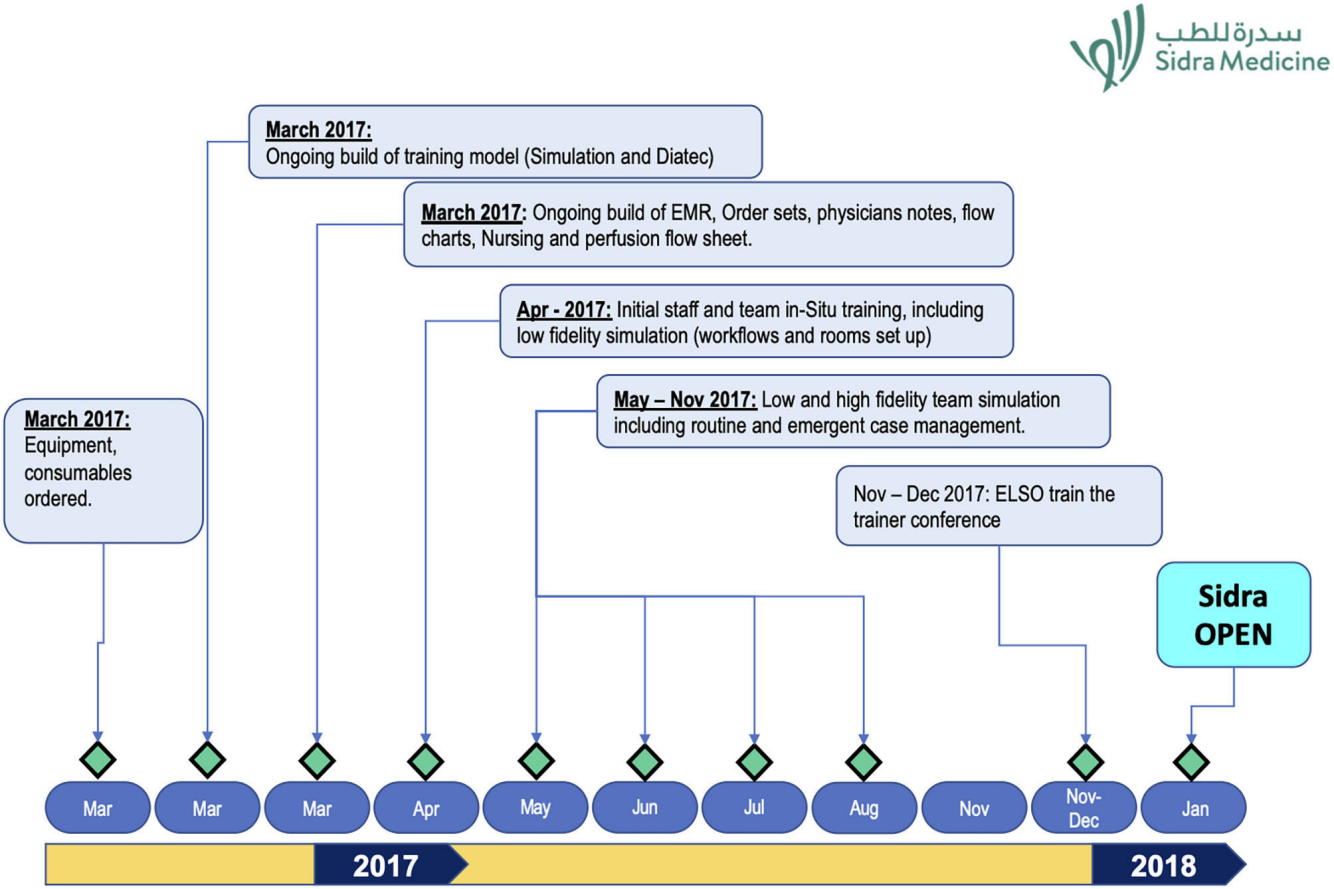
Sidra ECMO service milestones.

The timeline that was followed in setting up the program is shown in Figure 1.

## SIDRA ECMO Program

### Planning and Preparation

#### Institutional Commitment

The very beginning step in the development of any new program was an institutional commitment on the part of Sidra Medicine to develop a comprehensive ECMO program. The budget of the ECMO program was approved for building the program, and an additional yearly budget was allocated for consumable, staff hiring and training. It was agreed upon by everyone that starting such a program is pivotal for the country and the region.

#### ECMO Steering Committee

- We formed an ECMO steering committee, which included pediatrics surgery, pediatric cardiac surgery, neonatal intensive care unit (NICU), pediatric ICU ECMO physicians, perfusion, and ECMO nurses. Other allied health, pharmacy, radiology, blood bank, laboratory, and different pediatrics subspecialties were also required for the ECMO services as well as key individuals from the hospital administration to support the ongoing efforts. The ECMO program steering committee mission is to enhance the science of pediatric and adult critical care while providing advanced care to patients. There was a Medical ECMO Director as well as an ECMO coordinator who directed these efforts.

The scope of practice of the ECMO program steering committee included the following:

- The needs assessment to define the scope and role of the program at Sidra and within Qatar and the Middle East region, as well as identifying the patient population and the demand for the service, both immediate and in the future.
- Evaluation of necessary equipment and consumables.
- Identifying issues, addressing risks, and coordinating activities and timelines as well as operational planning needed for initiation of the clinical operations of the ECMO Service.
- Developing, implementing, and evaluating protocols, formalizing policies, and procedures in collaboration between its members, to facilitate evidence-based practices.
- Developing a multidisciplinary team to deliver, in a collaborative effort, the best family-centered patient care.
- Setting up and maintaining of ECMO to critically ill patients at Sidra Medicine within its vision for world-class service.
- The scopes of practice also included the setup and maintenance of Continuous Renal Replacement Therapy (CRRT) via the ECMO circuit at Sidra Medicine.
- Developing a robust advanced training and educational center for ECMO for not only the region but also internationally.
- Ensuring that patient statistics will be reported to the ELSO and compared to other international ECMO programs for benchmarking and improving quality.

#### The Needs Assessment

An essential step before setting up a new program was to assess its need and viability, which depends on the appropriate patient population, identifiable both clinically and geographically. The United Kingdom (UK) Collaborative ECMO Trial Group provided rigorous evidence of the cost-effectiveness of neonatal ECMO at 4 years for mature infants with severe but potentially reversible respiratory failure (8). As of January 2018, there are 30,884 neonatal respiratory ECMO runs, with 84% surviving ECLS and 73% survived to discharge (ELSO data) (2, 9). An epidemiological study from the United Stated showed that neonatal ECMO rate is 18 per 100,000 live births. Neonatal ECMO utilization increased, while mortality decreased during the study period (2002–2011) (10). The population of Qatar is nearing 2.9 million now, with the annual birth rate reaching close to 30,000 per year, so there was increasing demand for neonatal ECMO beds (10).

To measure the demand and need for this service locally, we reviewed the healthcare records and data from the existing neonatal ICU (Hamad General Hospital) in the country. There are 26,000 deliveries per year in Qatar. We looked at newborn infants who were admitted to the NICU who met the criteria to undergo ECMO therapy. The average number per year was identified to be between 12 and 16 per year. Of those, there was 8–10 severe diaphragmatic hernia per year, which required high-frequency oscillation and nitric oxide therapy. These cases in the past only have conventional treatment available and had a very high mortality rate, more than 80%.

We also anticipated increased demand for the ECMO services, as new advanced therapies will be offered at Sidra Medicine including cardiac surgeries in the neonatal period, outbreaks of severe bronchiolitis (11), or influenza (H1N1) (12–14) or other respiratory viruses (15). Furthermore, other indications such as pulmonary hypertension associated with severe chronic lung diseases in extreme preterm (16) and *ex-utero* intrapartum treatment (EXIT) procedures (12, 17), and referral from the region due to limited availability of service (18) would increase the need for ECMO.

#### Setting the Scope of Practice

The Steering Committee set the neonatal ECMO services according to the ELSO Guidelines for Neonatal Respiratory Failure. The ECMO program, in line with the Cardiovascular Center, embraced a multidisciplinary team approach to deliver exceptional care to patients treated at Sidra Medicine.

### Formation of ECMO Team

#### Key Personnel

The Physician–Neonatologist taking the role as activation director of the ECMO service formed a pivotal role in the process of the program development. The ECMO director would be responsible for the development of protocols and procedures to provide ECMO therapy efficiently and acting as the liaison between different services. He would supervise all processes and ensure monitoring of program quality. Individual leads for all the involved services were identified and coordinated.

#### Staffing Design

Care of the ECMO patient is directed and delivered by a collaborative effort of the multidisciplinary team under the umbrella of the ECMO steering committee, including the:

- ECMO Director
- ECMO Co-coordinator
- ECMO unit-specific medical lead
- ECMO Physicians in Neonatology
- ECMO Physicians in Pediatric Cardiac ICU
- ECMO Physicians Pediatric Intensive Care
- Pediatric Surgeons
- Cardiothoracic Surgeons
- Perfusionists,
- ECMO Specialists
- Registered Nurses and Registered Respiratory Therapists (RTs) in the NICU, PICU, and CICU.

ECMO-trained physicians and surgeons formed a core group of ECMO program along with the bedside treating team of intensive care physicians (neonatal, general pediatric, and cardiac). The team had a working knowledge and understanding of ECMO support and the technical ability for emergency troubleshooting and circuit intervention.

It was envisaged that policies, procedures, as well as clinical guidelines will be synchronized between all critical care units at Sidra Medicine. This will enable patients to receive the same level of service and evaluated by the same protocols and standards, irrespective of the physical location of their care.

#### ECMO Specialists Model

ECMO “Specialist” is the technical specialist with strong critical care background trained to manage the ECMO equipment and also the clinical needs of the patient on ECMO under the direction and supervision of an ECMO-trained physician (19, 20). The quality of care provided by an ECMO program is critically dependent on a team of ECMO specialists. After consideration of multiple models for care, the model of ECMO care where a RT/intensive care nurse works as a specialist was selected with backup and perfusionist help. This model was chosen because of the preexisting ECMO skill set of RT/ICU and has been studied as cost-effective (7). This also freed our perfusionists to spend more time in the cardiothoracic operating room. Implementation of an ECMO specialist-driven ECMO proved to be successful and was demonstrated by the minimal mechanical complications we had.

**Figure 2:**
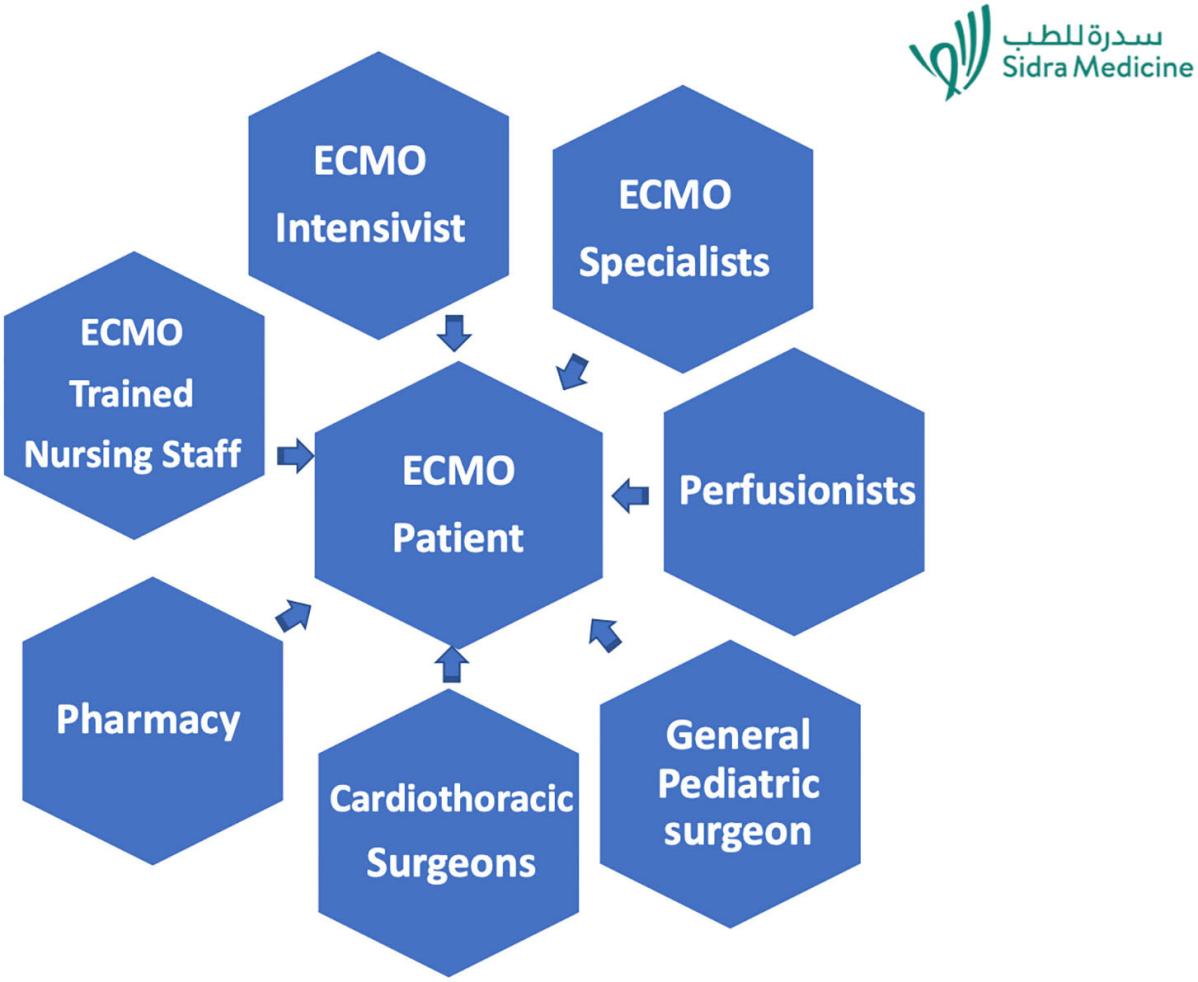
ECMO care model.

The ECMO specialist-based model is shown in Figure 2.

### Training of Staff

SIDRA recruited physicians and nurses with prior ECMO training from their previous workplace; however, among the team members, experience varied, and there was a need to build up a bigger team. To become clinically proficient, a comprehensive ECMO educational curriculum was developed to train subspecialty physicians, nurses, perfusionists, and RTs with minimal to no prior pediatric ECLS experience. The focus of this training was to provide an opportunity for dedicated practice of individual skills and separately to build and improve performance across multiple domains of expertise. Twenty ICU nurses from CICU/NICU and 10 RTs were chosen to undergo training. Of the 20 ICU nurses, 15 were novices and 5 had variable experience. Ten RTs had ECMO experience that varied from novice to advance. Neonatologists without much prior ECMO experience were trained gradually to be part of an ECMO team managed and led by a neonatologist with good prior ECMO experience. Both neonatal and cardiac ICU teams were trained simultaneously in the same training schedule; however, neonatal respiratory ECMO patients were decided to be managed separately in the neonatal ICU, necessitating establishment of a separate team.

### Preparation Phase

Once the equipment was procured, intense training sessions for a week by the ELSO team were followed by a 3-day Sophia Children's Hospital, Rotterdam visit to see Xenios machines in action to learn practical challenges. This hospital was established in 1863, and their ECMO program was commenced in 1996 so the services and teams were extremely experienced. Their protocols and workflows were reviewed, and troubleshooting was discussed. Moreover, the Xenios 3-day workshop from Germany was conducted for team training.

For new ECMO programs to achieve the designation as an ELSO center of excellence, they have to begin their journey with the path to excellence in life support. A pillar of attaining the quality care required for this is the presence of an established and institutionally supported ECMO education program.

A multistep educational program was delivered over 2 years to 30 ECMO team members, based on guidelines from the ELSO (19, 20). In the NICU team, 12 physicians were trained apart from an ECMO specialist.

The framework adopted by ELSO is the one that uses Miller's triangle. This is a framework for clinical assessment. It is a process of assessing clinical skills, competence, and performance.

ELSO recommends that each center develops training programs that accomplish the first three steps of Miller's triangle to allow for a successful implementation of the “Does” phase.

### Establishing ECMO Competency

The ECMO Medical Director and Coordinator/Manager were responsible for the training of the team, ensuring that:

1. Ongoing competency is maintained
2. Established guidelines and standards are defined in institutional policies and procedures

### ECMO Training Course Curriculum

The course content was planned to provide a consistent, effective multidisciplinary content with all providers exposed to a single curriculum for initial ECMO Training.

Additional training needs to be tailored to the specific needs of individual learners.

The ELSO-based educational program can be divided into three categories (details in Appendix I).

**Figure 3:**
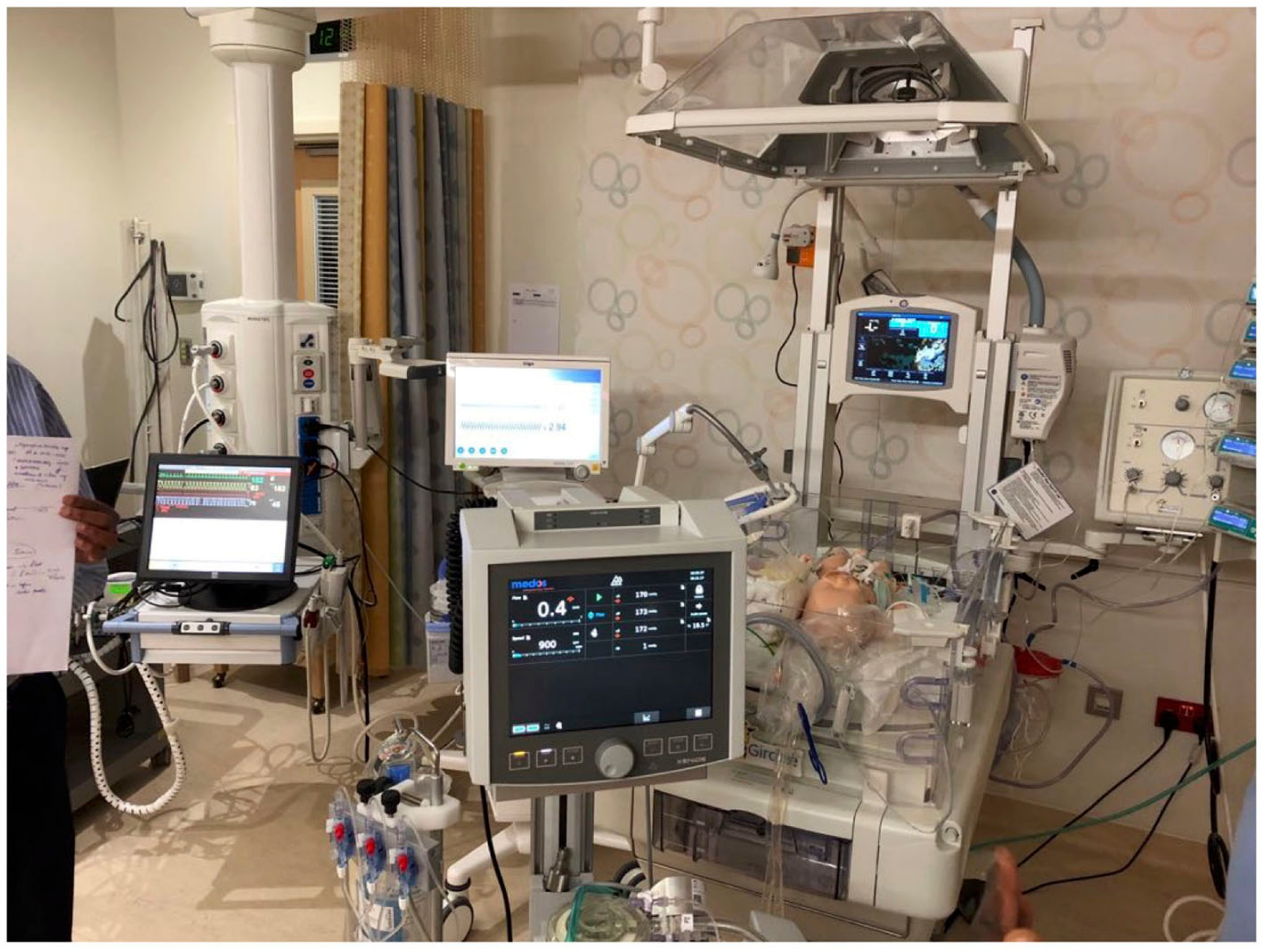
Wet lab training/simulation.

1. Knowledge-based didactic lectures: We ran multiple conferences that followed the ELSO guidelines for ECMO training and continuous education, which included indications for ECMO, contraindications, managing the patient on ECMO, cannulation strategy physiology, anticoagulation, and troubleshooting.
2. Practical Hands ON training basic wet labs and emergency drill with training for the change of different components (Figure 3).
3. High-fidelity simulation-based training on a modified neonatal manikin; participants were called to face, in small groups, in different critical scenarios, followed by debriefing time (Figure 3).

Record of training for individual ECMO specialists, nurses, and physician tracked as per the ELSO expectations and then final sign off for working in the ECMO team.

**Figure 4:**
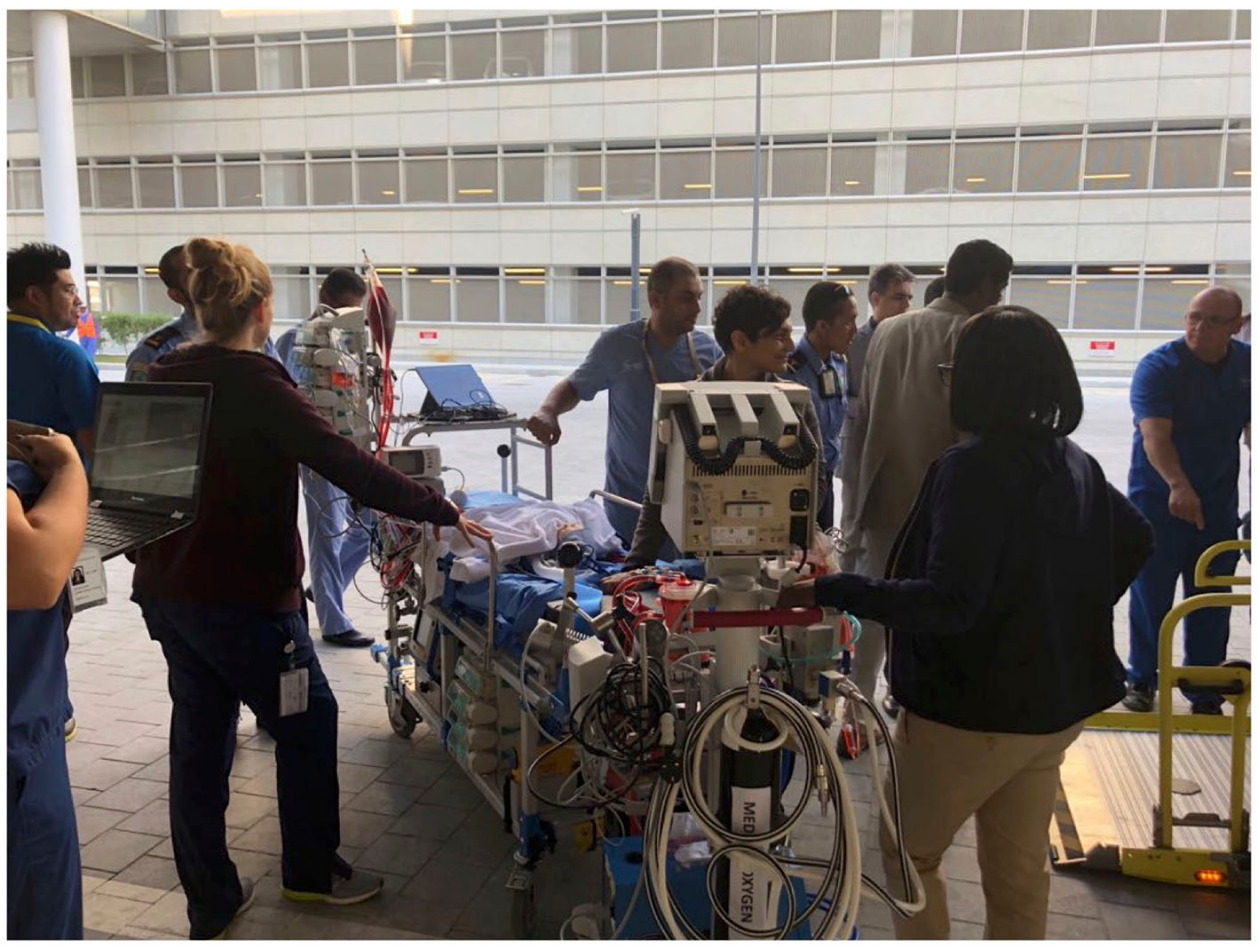
ECMO transport.

Simulation of ECMO transport specifically performed to gear up extramural and intramural transport on ECMO (Figure 4).

Simulation helped to build knowledge of ECMO support, and participants were also able to focus on vital technical and behavioral skills. Ongoing ECMO simulation will be used for quality improvement, clinical and educational research, and assessment or credentialing (21, 22).

Trainees underwent a pre-test for baseline competency assessment. Once the full training program is completed, a post-test was administered. Pre- and post-test scores were compared. Trainees rated the educational program through direct feedback.

### After Activation

Formal training received by the ECMO specialist and new physicians joining the team:

The majority of our ECMO specialists were encouraged to undergo a 1-week diploma course at La Petit Salpêtrière Hospital, Paris, France. Various members of the team have attended this weeklong ECMO diploma course. Moreover, they all attended the ELSO simulation training workshop in 2019 and received certificates. They all went through the ECMO transport workshop simulation spread over 2 days.

### Ongoing Credentialing

To keep up the knowledge and skills in a low-volume center, the ELSO teaching schedule being conducted in the hospital consists of:

- Monthly didactic lectures.
- Continuous weekly wet labs.
- Yearly knowledge-based and practical exams are conducted to evaluate training and learning.
- Annual ECMO conference at Sidra is conducted consisting of didactic, wet labs, and simulation.

#### Evaluation of Necessary Equipment and Consumables

While the protocols were being developed, the ECMO activation director, in cooperation with the ECMO steering committee, selected the program equipment. Equipment selection was based on the patient population as well as benchmarking alongside other institutions. We found our selection criteria on these critical factors (23):

1. Clinical efficiency.
2. Quality and safety.
3. Financial viability.

As a new minimized ECMO device, we used the Deltastream DP3 in combination with the HILITE 800, 2,400, and 7,000 LT (both Xenios Cardiopulmonary Solutions, Stolberg, Germany) oxygenator This ECMO equipment was approved recently by the FDA in the United States. Altogether, the different components built a very compact and easy-to-handle device with a weight of 5 kg. The system is entirely heparin-coated (Reoparine Medos AG) and has a priming volume of 275–600 ml depending on the oxygenator size. A recent study from seven European pediatric centers looked at their patient's outcomes on this device (24). They examined 233 patients whose median age was 1.9 (0–201) months. This new diagonal pump has demonstrated its efficacy in all kinds of mechanical circulatory and respiratory support with excellent outcomes. This DP3 diagonal pump is useful in generating physiological quality of pulsatile flow, without backflow into the circuit (25). The advantage of this system besides its small size and easy mobility was the only ECMO system, which is designed with fully integrated neonatal circuits and does not need any adaptation to fit in the pump head. It permits very low flows without a need for a bridge compared to other ECMO systems. These features became so apparent in our ECMO runs. We have transported our ECMO patients to a CT scanner, cardiac catheterization lab, and cardiothoracic operating room with ease and without any complications.

#### ECMO Program Planning and Milestones

We developed protocols and procedures for:

1. Technical backup, storage, and space.
2. Equipment safety and monitoring.
3. Maintenance and upkeep of point-of-care devices.
4. Robust methods of ensuring adequate supplies while avoiding the expiration of infrequently.
5. ECMO activation procedure.

#### Protocol Development

An extensive review of the literature was conducted, and visits to some of the hospitals that use the same equipment were conducted. We also leveraged the vast experience of our ECMO group; we drafted our protocols, which were reviewed by the members of our ECMO team including physicians, surgeons, nursing staff, ECMO specialists, perfusionists, and pharmacy. After every stakeholder input was incorporated, protocols were finalized.

We also have developed few programs embedded in our ECMO services including (a) neurocritical monitoring in collaboration with our neurology department, (b) CRRT therapy in collaboration with our nephrology department, (c) anticoagulation therapy in partnership with our hematology department, (d) ventilation management during ECMO, and (e) quality program, overseen by our quality department, and report to the ELSO. Medical management guidelines and protocols were written, and workflows for multidisciplinary team management were put in place.

### ECMO Program Activation and Evaluation

#### ECMO Program Activation

**Figure 5:**
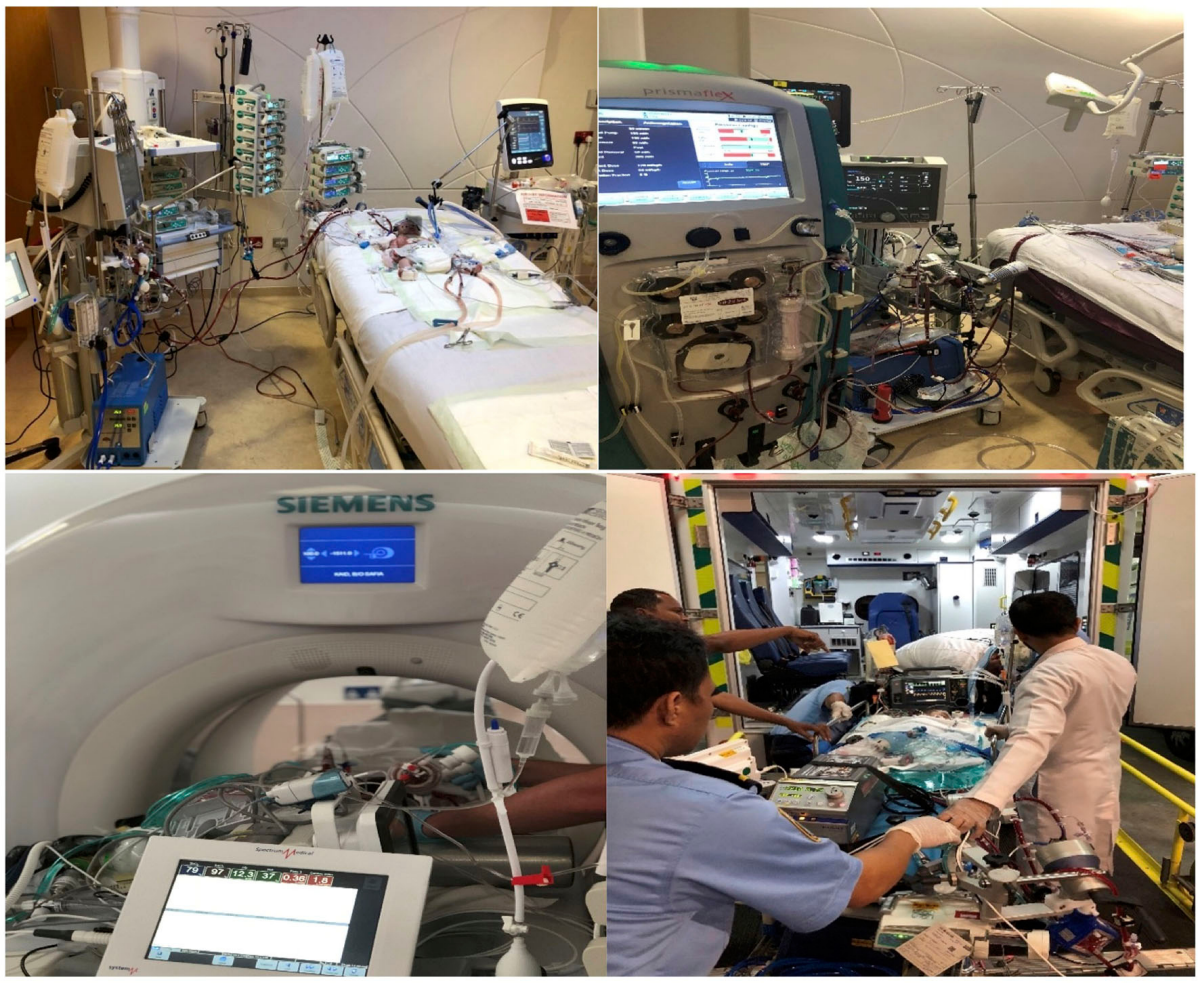
First ECMO patient.

Our ECMO program was activated in May of 2018 and first baby was referred for ECMO in June 2018 (Figure 5). Multiple factors played a considerable role in the successful launch of this program: (1) institutional commitment, (2) extensive comprehensive ECMO training program, (3) a committed ECMO team of individuals who pushed themselves hard to establish and improve the team daily, and (4) a formal consultative, multidisciplinary team to evaluate ECMO candidates.

#### Program Evaluation

The program is continuously monitored by the ECMO leadership team and our ECMO quality team. Meetings were scheduled monthly to discuss issues and review cases. Each case is discussed in detail with a goal to improve the quality of care.

As the ECMO program was getting established, both the NICU and PICU teams were trained simultaneously. Initially, a NICU ECMO specialist managed pediatric patients in the PICU as part of training and new neonatal ECMO physicians also participated in the care for these patients in PICU.

To evaluate the success of the program and for benchmarking, the ECMO database of Sidra Medicine Hospital was queried for all patients who received ECMO since activation (July 2018 to June 2019). All patients undergoing respiratory ECMO (both neonatal and pediatric) were included in this study.

### Patient's Selection for Neonatal ECMO

We followed the ELSO guidelines and developed a set of criteria based on a consensus process.

Consider and discuss ECMO in any near-term neonate: if unable to adequately oxygenate and/or ventilate despite maximal ventilator and cardiac support with reversible respiratory and/or cardiac failure. Any ECMO referral is channeled to a multidisciplinary team and evaluated by the intensivist, medical director, and specialists (cardiology, pediatrics, cardiothoracic surgery, etc.).

***The following Neonatal General inclusion and exclusion criteria(******26******)*** ***were followed:***

Gestational age, 34 weeks, or birth weight 2,000 g
No significant coagulopathy or uncontrolled bleeding
No major intracranial hemorrhage, Grade II or greater intraventricular hemorrhage
Reversible lung disease with the length of mechanical ventilation <10–14 days
No uncorrectable congenital heart disease
No lethal congenital anomalies
No evidence of irreversible brain damage
No evidence of irreversible multiple organ damage.

### Clinical Indications (27)

Neonates with severe respiratory failure, refractory to extensive medical management, with a potentially reversible etiology:

This may be indicated by:

1. Oxygenation Index >40 for >4 h
2. Oxygenation Index >20 with lack of improvement despite prolonged (>24 h) maximal medical therapy or persistent episodes of decompensation
3. Severe hypoxic respiratory failure with acute decompensation (PaO_2_ <40) unresponsive to intervention
4. Progressive respiratory failure and/or pulmonary hypertension with evidence of right ventricular dysfunction or continued high inotropic requirement.

Physical and laboratory parameters were collected prospectively, which allowed a retrospective analysis of the mechanical complications during ECMO runs. The Ethics Committee of Sidra Medicine waived ethical approval for publication of this retrospective analysis and the need for informed consent. All devices are approved for clinical use, no personalized data, and only routine laboratory parameters were used.

To assess the quality of the ECMO program, both neonatal and pediatric respiratory patients undergoing ECMO were evaluated.

### Statistical Analysis

We looked at all technical complications and benchmarked these numbers with five runs/year and the last 10 years of ELSO registry data (2009–2019) in the corresponding categories for comparison. Chi-square test was used to compare the categorical variables, and outcomes are presented in percentage, and *p*-values < 0.05 are considered significant.

## Results

Data were collected from the Sidra Medicine ECMO registry between July 2018 and May 2020 and also from the ELSO registry. A total of 27 patients have undergone ECMO therapy at Sidra Medicine in the first 22 months after establishing the program (July 2018 to May 2020), of which 11 patients (six newborn and five pediatric) who had respiratory indications were reviewed. The remaining patients had either cardiac indications or extracorporeal cardiopulmonary resuscitation (ECPR).

Six male and five female patients were treated with ECMO. Demographics data and pre-ECMO clinical status markers, which are also indications for ECMO, are described in Table 1. Primary diagnoses included meconium aspiration, CDH, ARDS, and pneumonia (Table 2). Dopamine and/or other cardiotonic agents were used in 96% of patients prior to ECMO. A total of 3,360 h of ECMO were run in 11 patients. The duration of ECMO averaged 305 h (range, 1 day to 7 weeks). Nine infants survived ECMO until discharge from the hospital.

**TABLE 1:** Demographic data for ECMO patients with survival outcome.

**Patient number**	**Age of commencing ECMO**	**Sex**	**Weight KG**	**Race**	**Diagnosis**	**Type of ECMO/ cannulation site**	**Days of ECMO**	**Outcome**
1	2 years	Male	13.3	African	Bacterial Pneumonia	V-V, RIJV	4	Survived
2	Day 1	Male	2.9	Middle Eastern	Severe PPHN	V-V, RIJV	1	Death
3	4 years	Female	20.3	Asian	Bacterial Pneumonia	V-V, RIJV	8	Survived
4	9 months	Male	7.98	Asian	RSV Pneumonia	V-V, RIJV	51	Survived
5	Day 1	Male	2.76	Middle eastern	CDH (Rt)	V-A, RIJV, RCCA	16	Death
6	Day 1	Male	2.8	Middle eastern	MAS,PPHN	V-V, RIJV	4	Survived
7	Day 1	Male	3.3	Asian	PPHN	V-V, RIJV	5	Survived
8	3 months	Female	2.12	African	Viral Pneumonia	V-V, RIJV	9	Survived
9	Day 1	Female	3.4	African	CDH (Rt)	V-V, RIJV	18	Survived
10	12 years	Female	23	African	Bronchiectasis	V-V, RIJV	8	Survived
11	Day 1	Female	3.48	African	MAS PPHN	V-V, RIJV	16	Survived

*V-V, Veno-venous; RIJV, Right Internal Jugular vein; RCCA, Right common carotid artery*.

**TABLE 1B:** Pre-ECMO settings at Sidra Medicine in included patients.

**Patient number**	**MODE of ventilation**	**Pre-ECMO respiratory support**							**Hemodynamic parameters and other relevant data**	
		**MAP**	**FiO_**2**_ (%)**	**Oxygen Index (OI)**	**pH**	**HCO_**3**_**	**Lactate**	**Nitric oxide (NO)**	**Mean BP mmHg**	**Inotropes/Other meds/Procedures**
1	HFO	24	100	55	7.22	17.4	6.2	yes	52	Epi, NorEpi, Milrinone, Vasopressin
2	HFO	20	100	50	7.02	7.6	14.2	yes	15	Dobutamine, Dopamine, Epi, Milrinone
3	HFO	15	100	42	7.21	27	1.0	No	53	Epi, Norepi
4	HFO	30	100	60	7.26	25.7	5.8	yes	69	Dopa, Epi, Narcotics, NMBAs
5	Conventional	18	100	61	7.21	21	1.4	yes	38	Epi, Narcotics
6	HFO	23	100	40.2	7.3	20	2.9	Yes	42	Dobut, Dopa, Epi, Milrinone, Narcotics, NMBAs, Bicarbonate
7	HFO	17	100	44.8	7.26	22.2	1.5	Yes	47	Narcotics, NMBA
8	HFO	28	100	75	7.13	14.5	4.8	No	58	Narcotics
9	HFO	17	100	43	7.13	14.5	4.8	Yes	54	Epi, Milrinone, Sildenafil, Narcotics
10	Conventional	15	100	51	7	18.2	1.1	Yes	65	Narcotics,NMBA
11	Conventional	18	100	47	6.9	7.6	8	Yes	47	Therapeutic Hypothermia, Surfactant, Narcotics, Epinephrine

*HFOV, High Frequency Oscillatory; MAP, Mean Airway Pressure; Epi, epinephrine; NorEpi, Nor-Epinephrine; Dopa, dopamine; Dobut, Dobutamine; NMBA, Neuromuscular blocking agents*.

**TABLE 2:** SIDRA ECMO respiratory outcomes data (July 2018–May 2020) ELSO centers registry data (2009–2019) of neonatal and pediatric group.

**Neonates-Respiratory**					
**Diagnosis**	**At my center (SIDRA)** ***n*** **=** **6 (%)**	**ELSO** **≤** **5 runs/year** ***n*** **=** **336 (%)**	**Total ELSO registry** ***n*** **=** **5,478 (%)**	***P*****-value (5 runs/year)**	***P*****-value (ELSO total)**
CDH	2 (33)	115 (34)	2,285 (42)	0.95	0.65
MAS	1 (17)	113 (33.6)	1,547 (28)	0.39	0.58
PPHN/PFC	2 (33)	89 (26.4)	1,375 (25)	0.71	0.65
Sepsis	1 (17)	19 (5.6)	271 (05)	0.23	0.18
**Pediatric-Respiratory**					
**Diagnosis**	**At my center (SIDRA)** ***n*** **=** **5 (%)**	**ELSO** **≤** **5 runs/year** ***n*** **=** **108 (%)**	**Total ELSO registry** ***n*** **=** **1,185 (%)**	***P*****-value (5 runs/year)**	***P*****-value (ELSO total)**
Asthma	0 (0)	10 (9.25)	94 (08)	0.48	0.51
Bacterial pneumonia (2); Bronchiectasis (1)	3 (60)	42 (38.88)	306 (25)	0.35	0.07
Pertussis	0 (0)	3 (2.77)	63 (05)	0.71	0.26
Viral pneumonia	2 (40)	53 (49.07)	722 (60)	0.70	0.83

*CDH, Congenital Diaphragmatic Hernia; MAS, Meconium Aspiration Syndrome; PPHN, Persistent Pulmonary Hypertension of Newborn; PFC, Persistent Fetal Circulation*.

### Mechanical Complications

There were only two mechanical complications noted in these 11 ECMO runs, which include one canula issue (clot) and one circuit component clot, but zero pump failure, oxygenator failure, or pump clots (Table 3).

**TABLE 3:** Benchmarking of mechanical complications in SIDRA ECMO program against other ELSO centers registry data of neonatal and pediatric group.

**Neonatal-Respiratory**					
**Mechanical complication**	**At my center (SIDRA)** ***n*** **=** **6 (%)**	**ELSO** **≤** **5 runs/year** ***n*** **=** **493 (%)**	**Total ELSO registry** ***n*** **=** **7,366 (%)**	***P*****-value (≤5 runs/year)**	***P*****-value (ELSO total)**
Oxygenator failure	0 (0)	27 (5.5)	324 (4)	0.55	0.62
Raceway rupture	0 (0)	4 (0.81)	12 (0.2)	0.8	0.92
Other tubing rupture	0 (0)	2 (0.41)	24 (0.32)	0.87	0.89
Pump Failure	0 (0)	9 (1.8)	83 (1.1)	0.74	0.82
Heat exchanger malfunction	0 (0)	0 (0)	22 (0.3)	0.89	0.90
Clots: hemofilter	0 (0)	9 (1.8)	345 (4.6)	0.74	0.3
Clots: Circuit Component	0 (0)	189 (38.3)	2,705 (36)	0.06	0.07
Air in circuit	0 (0)	21 (4.3)	262 (3.5)	0.6	0.64
Crack in pigtail connectors	0 (0)	6 (1.21)	52 (0.7)	0.78	0.83
Cannula Problems	1 (16.6)	48 (9.74)	893 (12)	0.57	0.7
Circuit change	0 (0)	17 (3.5)	159 (2.1)	0.64	0.74
Clots and air Emboli	0 (0)	1 (0.2)	5 (0.06)	0.9	0.9
Thrombosis/Clots: Circuit component	0 (0)	8 (1.6)	45 (0.6)	0.75	0.85
**Pediatric-Respiratory**					
**Mechanical complication**	**At my center (SIDRA)** ***n*** **=** **5 (%)**	**ELSO** **≤** **5 runs/year** ***n*** **=** **358 (%)**	**Total ELSO registry** ***n*** **=** **4,773 (%)**	***P*****-value (≤5 runs/year)**	***P*****-value (ELSO total)**
Oxygenator failure	0 (0)	29 (8.1)	300 (6)	0.5	0.60
Raceway rupture	0 (0)	0 (0)	5 (0.1)	-	0.94
Other tubing rupture	0 (0)	1 (0.3)	11 (.2)	0.9	0.92
Pump Failure	0 (0)	8 (2.2)	60 (1)	0.74	0.82
Heat exchanger malfunction	0 (0)	2 (0.56)	11 (.2)	0.87	0.92
Clots: hemofilter	1 (20)	12 (3.35)	223 (5)	0.70	0.67
Clots: Circuit Component	0/ (0)	66 (18.4)	1,434 (30)	0.29	0.14
Air in circuit	0 (0)	22 (6.1)	244 (5)	0.57	0.6
Crack in pigtail connectors	0 (0)	4 (1.1)	48 (1)	0.81	0.82
Cannula Problems	0 (0)	32 (8.9)	663 (14)	0.48	0.36
Circuit change	0 (0)	12 (3.35)	147 (3)	0.67	0.70
Clots and air Emboli	0 (0)	0 (0)	15 (.3)	-	0.90
Thrombosis/Clots: Circuit component	0 (0)	2 (0.59)	39 (.8)	0.86	0.84

### Clinical Complications

In the newborn group, there were two neurological complications and one renal failure needing renal replacement therapy, as listed in Table 4. In the pediatric group, there was only one pulmonary complication, which was pneumothorax. There were no mechanical or clinical complications reported during transport on ECMO.

**TABLE 4:** Benchmarking of Clinical complications in SIDRA ECMO program against ELSO registry data.

**Complications in Neonatal patients (*n* = 3)**	**At my Center (SIDRA) Total Neonates = 6 (%)**	**ELSO ≤ 5 runs/year *n* = 493 (%)**	**Total ELSO Registry *n* = 7,366 (%)**	***P*-value (5 runs/year)**	***P*-value (ELSO total)**
Neurologic: Seizures confirmed by EEG	1 (16.6)	9 (1.8)	234 (3.2)	0.009	0.06
Neurologic: CNS infarction (US or CT or MRI)	1 (16.6)	12 (2.4)	234 (3.2)	0.03	0.06
Renal: Renal Replacement Therapy required	1 (16.6)	91 (18.4)	2,198 (30)	0.91	0.5
**Complications in Pediatric patients (*****n*** **=** **1)**	**At my Center (SIDRA) Total Pediatrics** **=** **5 (%)**	**ELSO** **≤** **5 runs/year** ***n*** **=** **358 (%)**	**Total ELSO Registry** ***n*** **=** **4,773 (%)**	***P*****-value (5 runs/year)**	***P*****-value (ELSO total)**
Pulmonary: Pneumothorax requiring treatment	1 (20)	18 (5.1)	407 (8.5)	0.10	3.5

## Discussion

This original paper is the first ever to investigate the process of activation and success of a first regional neonatal ECMO program in the Middle East. There are existing adults and pediatric ECMO services in the other countries in the Middle East region and even in Qatar; however, these services were underutilized for the neonatal respiratory ECMO and were mainly limited to Cardiac ECMO. As per our knowledge, there is no such similar program in the Middle Eastern region which is led and managed by neonatologists. The demography and social structure of the Middle Eastern population are different as compared to other parts of the world. The growing population may create a need for the future establishment of more ECMO programs in the Middle Eastern region, and this experience may help them. This was an ECMO specialist-driven program that was executed in a very planned manner over a period of time with rapid ramping up once the hospital was opened. The key feature of the success of program implementation was outcomes comparable to (albeit with minimal mechanical complications) the ELSO registry despite setting it up on a Greenfield site. This can be attributed to careful planning with systematic execution, collaborating available experience, and extensive training of ECMO staff before activation. Although the number for comparison was small, the clinical outcomes were also comparable to the ELSO registry, including the percentage of babies discharged from the hospital. There were no patient complications that occurred during transport, demonstrating that adequate training and proper protocols can execute safe transport in a new system.

We have excellent outcomes in this group when compared to other low-volume center data. We appreciate that we had two clot complications in 11 patients we reported, which may seem high compared to ESLO (although not statistically different). We have reviewed and discussed our anti-coagulation protocol among all multidisciplinary teams including hematology, and we are regularly auditing this so that these complications will not occur in the future. Clinical complications were less difficult to conclude due to the small number size, and we shall evaluate this regularly.

**There were particular challenges faced during ECMO activation, which were as follows:**

1. Sidra ECMO program was initially built with combined teams from NICU and PICU but eventually separated into two independent programs; one is offering neonatal respiratory ECMO in the NICU. Getting ECMO specialists, staffing the team, and ensuring ECMO competency of the novice nurses were very challenging. Also, ECMO physicians had mixed experience, with some having extensive training and others with limited experience. Due to the high demand for in-house physician cover, extra commitment was needed to get trained and improve skills for ECMO physicians. A separate ECMO roster was putting other neonatologists in the NICU team under pressure and it needed their flexibility to support the increasing needs of the program.
2. Allocation of time during the training to train people from different specialties was also a challenge.
3. Although ECMO experienced staff were recruited, new machines, different terminologies, and unfamiliarity with new pumps resulted in a slow learning curve, with pumps failing twice during training sessions with even breakdown of the circuit. So, getting everyone aligned on the same process was an ongoing challenge, which was tackled with bedside one-to-one teaching to ECMO specialists.
4. Laboratory support running ECMO coagulation parameters in the lab is not a usual practice for non-ECMO centers. Our hematology department developed and standardized all the ECMO critical testing required, including anti-X essays.
5. ECMO transport program—transferring a baby on ECMO from a referring hospital and also for procedures like CT scanning—had a lot of logistical challenges with extra training needed for the staff in the radiology department and the ECMO team. Now, with training additional staff, our ECMO transport team covers the whole Qatar region and aspires to do international transport very shortly.

## Conclusion

The article summarizes the process and evaluation of setting up a new ECMO program on a Greenfield hospital site. It can be a guide for future hospitals trying to establish a similar program. The development of neonatal ECMO programs was desperately needed in the Middle East region and was achieved successfully. Although we have minimal patient data, the number was achieved in a short duration of 18 months, and the complication rates were lower than or comparable to ELSO data, assuring a successful implementation. The number of patients is a limitation in this study and no real conclusions can be drawn regarding patient outcomes. However, the key components in building a successful ECMO program appear to be institutional commitment, multidisciplinary leadership, and organized training. Using consistent and continuous training is the key to the success of the ECMO specialist's model.

## Data Availability Statement

The datasets generated for this study are available on request to the corresponding author.

## Ethics Statement

Written informed consent was obtained from the individuals for the publication of any potentially identifiable images or data included in this article.

## Author Contributions

ME conceptualized and drafted the manuscript. KM extracted ECMO data and edited and finalized the manuscript. SY helped in reviewing and analyzing data. DA made substantial role in developing, running this new ECMO program with active role in managing patients on ECMO. He has taken over ECMO lead position and reviewed and corrected the revised manuscript before submission. All authors contributed to the article and approved the submitted version.

## Conflict of Interest

The authors declare that the research was conducted in the absence of any commercial or financial relationships that could be construed as a potential conflict of interest.

## Abbreviations

AHA: American Heart Association
AKI: acute kidney injury
CDH: congenital diaphragmatic hernia
MAS: meconium aspiration syndrome
PPHN: persistent pulmonary hypertension of newborn
PFC: persistent fetal circulation
TGA: transposition of great arteries
DORV: double outlet right ventricle
CPR: cardiopulmonary resuscitation
CT: computed tomography
ECLS: extracorporeal life support
ECMO: extracorporeal membrane oxygenation
ECPR: extracorporeal cardiopulmonary resuscitation
EEG: electroencephalographic
ELSO: Extracorporeal Life Support Organization
ICU: intensive care unit
MRI: magnetic resonance imaging
NICU: neonatal ICU
OR: odds ratio
PHTN: pulmonary hypertension
PICU: pediatric ICU.
