# Supplementary material for: Successful Establishment of the First Neonatal Respiratory Extracorporeal Membrane Oxygenation (ECMO) Program in the Middle East, in Collaboration With Pediatric Services
Source: Front Pediatr. 2020 Sep 11;8:506. doi: 10.3389/fped.2020.00506 (PMC7516255; doi:10.3389/fped.2020.00506)
Supplement: Supplementary file 1 [file Data_Sheet_1.docx]

**APPENDIX I : ECMO training Course curriculum**

Course content should provide a consistent, effective multidisciplinary content with all providers exposed to a single curriculum for initial ECMO Training.

Additional training need to be tailored to the specific needs of the individual learners.

**DIDACTIC (CLASSROOM) COURSE**

1. Introduction to ECMO
   1. History
   2. Current status
   3. Risks and benefits
   4. Membrane gas exchange physics and physiology
   5. Oxygen content, delivery and consumption
   6. Shunt physiology
   7. Types of ECMO
   8. Future applications
   9. Research
   10. ELSO Registry
2. Physiology of Diseases treated with ECMO
   1. Respiratory failure (eg Pneumonia, aspiration pneumonitis, ARDS, Pulmonary embolism etc)
   2. Pulmonary embolism
   3. Sepsis
   4. Postcardiotomy
   5. Heart transplantation
   6. Cardiomyopathy and myocarditis
   7. Other
3. Pre-ECMO procedures
   1. Notification of the ECMO Team
   2. Selection of catheters
   3. VV or VA
   4. Cannulation procedures (open, Percutaneous)
   5. Initiation of ECLS
   6. Responsibilities of team Members
4. Criteria for ECMO
   1. Patient selection criteria
   2. Pre-ECMO evaluation
   3. Contraindications
5. Physiology of Venoarterial and Venovenous ECMO
   1. Vessel cannulation (VA, VV, VAV)
   2. Physiology
   3. Advantages and disadvantages
6. Blood products and Coagulation
   1. Blood products and interactions
   2. Coagulation cascade
   3. Blood surface interactions and coatings
   4. Heparin pharmacology
   5. SIRS
   6. Laboratory anticoagulation monitoring studies
   7. Amicar, Protamine, recombinant clotting factors
   8. Disseminated intravascular coagulation
7. ECMO Equipment and Circuit Components
   1. ECMO Circuit design
   2. ECMO circuit components (cannula, compliance chamber, pump, venous return monitor, in-line saturation monitor, pressure monitor, heater, hemofilter, bubble detector, flow meter)
   3. Oxygenator function and blood gas control
   4. ECMO supply cart
   5. Other ECLS equipment (CVVH, Plasmapheresis, etc)
8. Cannulation and Initiation of ECMO support
   1. Circuit priming
   2. Preparing the patient
   3. Initiating ECLS support
9. Daily Patient and Circuit management
   1. Patient
      1. Bedside care of the ECMO patient (e.g. ambulation)
      2. Fluids, electrolytes and nutrition
      3. Respiratory (e.g. Ventilator management, tracheostomy, extubation , etc)
      4. Neurologic
      5. Infection control
      6. Sedation and pain control
      7. Hematology
      8. Cardiac
      9. Psychosocial
      10. Pharmacologic issues
      11. Lab Schedule
      12. Documentation and orders
   2. Circuit
      1. Aseptic technique
      2. Pump and gas flow
      3. Pressure monitoring
      4. Blood products infusion techniques
      5. Circuit infusions
      6. Management of anticoagulation
      7. Circuit checks
      8. Hemofilteration setup
10. Emergencies and complications during ECMO
    1. Medical
       1. Intracranial and other hemorrhage
       2. Pneumothorax and pneumopericardium
       3. Cardiac arrest
       4. Arrhythmias
       5. Hypotension and hypovolemia
       6. Hypertension
       7. Severe coagulopathy
       8. Seizures
       9. Hemothorax and Hemopericardium
       10. Uncontrolled bleeding
       11. Electrolyte imbalance
       12. Renal failure
    2. Mechanical
       1. Circuit disruption
       2. Raceway rupture
       3. Cavitation
       4. System or component alarm and failure (pump, compliance chamber, venous return monitor, oxygenator, heater, flow sensor)
       5. Air embolus
       6. Inadvertent decannulation
       7. Clots
11. Management of Complex ECMO cases
    1. Surgery on ECMO
    2. Postoperative bleeding
    3. Transport on ECMO (inter / intrahospital)
12. Weaning from ECMO
    1. Techniques and complications
    2. Clinical indications of pulmonary and cardiac recovery
    3. Pump and gas flow weaning techniques
    4. Ventilator changes during weaning
    5. Trial off
    6. Decannulation and low flow
13. Decannulation
    1. Personnel needed
    2. Medications required
    3. Potential complications
    4. Vessel ligation
    5. Vessel reconstruction
    6. Percutaneous approach
    7. Post –ECMO complications
14. Outcome of ECMO patients
    1. Short and long term outcomes
    2. Institutional followup protocol
    3. Literature review
15. Ethical and social issues
    1. Consent process
    2. Parental and Family support
    3. Withdrawal of ECMO support

Training should be focused on site specific ECMO techniques and all ECMO team members must obtain a thorough understanding of ECMO equipment and circuit design used at Sidra.

HANDS ON TRAINING

1. Equipment: component and function check
   1. Circuit Check
      1. Tubing
      2. Sample ports
      3. Pump head
      4. Pump control and alarm
      5. Pressure monitoring
      6. Servo regulation panel
      7. Oxygenator
      8. Sweep gas monitoring
      9. Heat exchanger
      10. Water Heater
      11. Other (bridge, compliance chamber, flow sensor, arterial filter, bubble detector, etc)
   2. Basic Procedures
      1. Blood sampling
      2. Bedside ACT checks
      3. Pigtail and stopcock changes
      4. Blood products administration
      5. IV infusion and medication administration
      6. ECLS documentation
      7. ECMO order set review
      8. Other ( roller pump head occlusion checks)
   3. Emergency Procedures
      1. Clamping off ECMO
      2. Massive blood loss from circuit
      3. Tubing replacement
      4. Oxygenator failure
      5. Air in circuit and de-airing circuit
      6. Loss of venous return
      7. Inadvertent decanulation
      8. Power failure
      9. Pump head failure
      10. Circuit and / or component change

WATER DRILLS

HIGH FIDELITY SIMULATION
